# Electrospun polysuccinimide scaffolds containing different salts as potential wound dressing material

**DOI:** 10.3762/bjnano.15.65

**Published:** 2024-07-02

**Authors:** Veronika Pálos, Krisztina S Nagy, Rita Pázmány, Krisztina Juriga-Tóth, Bálint Budavári, Judit Domokos, Dóra Szabó, Ákos Zsembery, Angela Jedlovszky-Hajdu

**Affiliations:** 1 Laboratory of Nanochemistry, Institute of Biophysics and Radiation Biology, Semmelweis University, Nagyvárad tér 4, 1089, Budapest, Hungary; 2 Institute of Medical Microbiology, Semmelweis University, Nagyvárad tér 4, 1089, Budapest, Hungary; 3 Department of Oral Biology, Semmelweis University, Nagyvárad tér 4, 1089, Budapest, Hungary

**Keywords:** antibacterial activity, electrospinning, polysuccinimide, scaffold, wound dressing

## Abstract

In this research, we applied electrospinning to create a two-component biodegradable polymeric scaffold containing polysuccinimide (PSI) and antibacterial salts. Antibacterial agents for therapeutical purposes mostly contain silver ions which are associated with high environmental impact and, in some cases, may cause undesired immune reactions. In our work, we prepared nanofibrous systems containing antibacterial and tissue-regenerating salts of zinc acetate or strontium nitrate in different concentrations, whose structures may be suitable for developing biomedical wound dressing systems in the future. Several experiments have been conducted to optimize the physicochemical, mechanical, and biological properties of the scaffolds developed for application as wound dressings. The scaffold systems obtained by PSI synthesis, salt addition, and fiber formation were first investigated by scanning electron microscopy. In almost all cases, different salts caused a decrease in the fiber diameter of PSI polymer-based systems (<500 nm). Fourier-transform infrared spectroscopy was applied to verify the presence of salts in the scaffolds and to determine the interaction between the salt and the polymer. Another analysis, energy-dispersive X-ray spectroscopy, was carried out to determine strontium and zinc atoms in the scaffolds. Our result showed that the salts influence the mechanical properties of the polymer scaffold, both in terms of specific load capacity and relative elongation values. According to the dissolution experiments, the whole amount of strontium nitrate was dissolved from the scaffold in 8 h; however, only 50% of the zinc acetate was dissolved. In addition, antibacterial activity tests were performed with four different bacterial strains relevant to skin surface injuries, leading to the appearance of inhibition zones around the scaffold discs in most cases. We also investigated the potential cytotoxicity of the scaffolds on human tumorous and healthy cells. Except for the ones containing zinc acetate salt, the scaffolds are not cytotoxic to either tumor or healthy cells.

## Introduction

Polymer mixtures contain at least one component besides the polymer, such as nanoparticles, inorganic salts, or other polymers. These types of polymer mixtures are used for medical purposes; for example, metronidazole/poly(ε-caprolactone) (PCL)/alginate for dental implants or poly(lactic acid)/hydroxyapatite in orthopedics [1–2]. Biocompatible polymers are widely used in biomedical fields, such as stents, drug delivery systems in cancer therapy, bone repair, dentistry, joint prostheses, and tissue engineering [2–6]. Polymers have several advantageous properties for these applications as they are flexible, variable in weight, have excellent biocompatibility, are resistant to biochemical degradation, and have suitable physical and mechanical characteristics. Moreover, they can be formed to the desired shape [6]. It is also worth mentioning that different drugs or other additives can be easily linked to the polymer or distributed to them in the same solvent; thus, a multicomponent composite system can be generated. Based on the parameters mentioned above, wound-dressing application is one of the most relevant research areas for these systems [7].

An effective wound dressing should fulfill multiple needs: shielding the wound against bacterial infection, facilitating proper gas exchange, providing an environment that promotes healing, and controlling biofluid production [8–9]. Furthermore, it should be nontoxic and hypoallergenic [10].

By using electrospinning, nanofibers can be created and used in numerous biomedical applications, such as tissue engineering, wound dressing, and drug delivery [11–12]. Electrospinning has many advantages: it is a simple technique, cost-effective, reproducible, scalable, and reliable. In addition, various polymers can be used as starting material, and fibers can be produced with uniform diameters in a controlled way [13–14]. Electrospun fibers are similar to macromolecule networks (e.g., collagen, fibrinogen, elastin) around the cells, which are called extracellular matrix (ECM). The ECM has fiber diameters in the size range of 50–500 nm and has several functions in the human body, including wound healing [15–16]. Nanofibers produced by electrospinning have beneficial structural attributes, such as elevated porosity, high specific surface area, and nanoscale fiber dimensions; thus, adequately mimicking the ECM and promoting cellular adhesion, growth, proliferation, and differentiation. The electrospinning technique offers the possibility of using the formed scaffold as a wound dressing with fibers of proper size and morphology. The porous nature of the scaffold enables the drainage of wound fluids and facilitates the entry of oxygen from the atmosphere [17].

To achieve an antibacterial effect, one option is the introduction of salts to the polymer solution. The addition of salts to the electrospinning solution leads to changes in the conductivity, viscosity, shearing strength and morphology, and fiber diameter of the prepared scaffolds [18–21]. The antibacterial effectiveness of the fibrous structure is significantly influenced by incorporating salts or nanoparticles. When electrospun fibers are combined with inorganic nanoparticles [22], they can become resistant to bacteria. However, their ability to enhance antibacterial properties is limited due to the encapsulation of certain nanoparticles within the fibers [23]. One of the most common approaches is the introduction of antibiotics. However, with the misuse and/or overuse of these types of drugs, there is the risk of antibiotic resistance (AMR), which is one of the top-ten global public health threats, according to the World Health Organization [24]. A 2022 cross-sectional study in Hungary revealed that multidrug-resistant (MDR) bacterial infections caused extended hospital stays and increased patient mortality [25]. One option to solve this problem could be to apply silver ions, which are frequently used in clinical practice. However, they can cause a large environmental burden [26–27] and, in some cases, allergies [28]. Since urgent actions are needed to stop AMR and avoid the negative environmental impact, developing other agents and treatment strategies to fight bacterial pathogens with similar effects to antibiotics and silver is essential. In our study, inorganic salts, namely Zn(O_2_CCH_3_)_2_ and Sr(NO_3_)_2_, were added to the polymer fibers. These salts possess antibacterial properties and stimulate cell proliferation as well as tissue regeneration [29–32].

Based on the special requirements to fulfill as a wound dressing material, such as biocompatibility, biodegradability, good gas permeability, and water retention capacity, polysuccinimide (PSI) was used for the preparation of electrostatic fibers. Polysuccinimide is a nontoxic [33], biocompatible, and biodegradable polymer. Based on the literature, it is extensively researched for biomedical applications [21,33–43]. It is also known that PSI turns into polyaspartic acid (PASP) at physiological conditions, which is a degradable biomaterial [24–25,36–37].

This study aimed to create a two-component polymer-composite scaffold as a potential wound dressing material by electrospinning, using antibacterial salts (Zn(O_2_CCH_3_)_2_ or Sr(NO_3_)_2_) in addition to PSI. We performed the following experiments: physical and chemical characterization of the fibers by scanning electron microscopy (SEM), Fourier-transform infrared spectroscopy (FTIR), mechanical tests, investigation of salt dissolution from the scaffolds, examination of their antibacterial activity against four different bacterial strains (*Escherichia coli*, *Pseudomonas aeruginosa*, *Bacillus subtilis*, and *Staphylococcus epidermidis*), and finally cytotoxicity assays on two human cell types (i.e., MG-63 tumor cell line and 155BR fibroblasts).

## Materials and Methods

*N*,*N*-Dimethylformamide (DMF ≥99.5%, Alfa Aesar, USA); dimethylsulfoxide (DMSO ≥99.9%, Fisher Chemical, USA); zinc acetate (99,99%, Sigma-Aldrich, USA); strontium nitrate (Reanal, Hungary); ʟ-aspartic acid (99%, Amresco, USA); phosphoric acid (≥99.0%, Sigma-Aldrich, USA); *E. coli* (ATCC 25922); *P. aeruginosa* (ATCC 27853); *B. subtilis* (ATCC 6633); *S. epidermidis* (ATCC 14990); Mueller–Hinton agar (Biolab Zrt., Hungary); minimum essential medium (MEM, Gibco, USA); Dulbecco's modified Eagle medium (DMEM, Gibco, USA); MEM, no glutamine, no phenol red (Gibco, USA); phosphate-buffered saline (Gibco, USA); fetal bovine serum (FBS, Gibco, USA); penicillin/streptomycin mix (Gibco, USA); ʟ-glutamine (Gibco, USA); non-essential amino acid solution (NEAA, Gibco, USA); trypsin/EDTA solution (Sigma-Aldrich, USA); cell proliferation reagent WST-1 (Roche, Switzerland); and ultra-purified water (Zineer Power I Water Purification System). All reagents were used without any further purification.

### Polysuccinimide synthesis

The PSI was produced by thermal polycondensation in the presence of a phosphoric acid catalyst [2]. Phosphoric acid (20 g) and ʟ-aspartic acid (20 g) were mixed in a round-bottom flask and placed into a rotatory vacuum evaporator (RV10, digital rotary evaporator, IKA, Germany) with a rotation speed of 130 rpm. The temperature was gradually changed from room temperature to 180 °C, and the pressure was gradually reduced to 3 mbar. The synthesis lasted 8 h. The DMF was added to the synthesized polymer, stirred at 80 rpm, and left to dissolve for 60 min. The solution was dripped into distilled water, precipitating the PSI as a pellet. The pellet was filtered and washed using a vacuum filter to remove the phosphoric acid catalyst and the unreacted ʟ-aspartic acid monomeric molecules. Then, the pellet was mixed in distilled water for 10 min. This step was repeated until the pH of the filtrate changed from acidic to neutral. Finally, the PSI powder was dried at 40 °C and stored at room temperature. Based on our previous publications, the molecular weight of the polymer is 28 500 ± 3000 Da [44].

### Preparation and characterization of the solutions

The preparation of solutions for electrospinning was the following: a 25% (w/w) mixture was made from 1 g of PSI powder and 3 g of DMF. The solution was mixed using a magnetic stirrer (100 rpm) for one day to dissolve. The different amounts of salts were added to the PSI/DMF solution and mixed for one day. The weights of the salts, the DMF, and the PSI in the solutions are listed in Table 1.

**Table 1:** The components of the polymer/salt solutions and the applied voltages and flow rates at the electrospinning in the case of the PSI and different PSI + salt samples. The numbers before the polymer and the salts mean % (w/w). All experiments were performed at room temperature.

Sample name	PSI/DMF (g)		PSI/DMF + salt (g)						

	PSI (g)	DMF (g)	DMF (g)	salt (g)	entire mass (g)	viscosity (Pas)	conductivity (μS/cm)	voltage (kV)	flow rate (mL/h)

25 PSI	1	3	—	—	—	1.68	57.37	15	0.5
20 PSI + 5 Sr(NO_3_)_2_	1	3	0.75	0.25	5	0.899	746.7	19.1	0.5
20 PSI + 7.5 Sr(NO_3_)_2_	1	3	0.625	0.375	5	1.14	1572	16	0.4
20 PSI + 10 Sr(NO_3_)_2_	1	3	0.5	0.5	5	2.15	1843	16.7	0.4
20 PSI + 5 Zn(O_2_CCH_3_)_2_	1	3	0.75	0.25	5	1.14	101.5	15.2	0.5

The conductivity of the solution was determined with the SevenCompact Duo S213 Benchtop pH/mV/Conductivity Meter Cond Sensor InLab^®^ 710 (Mettler Toledo, USA) and the viscosity with the Sine-wave Vibro Viscometer SV-10 **(**A&D Company, Limited, Japan). The conductivity and viscosity values of the solutions are in the Table 1.

### Electrospinning

The PSI/DMF/salt solutions were placed in a syringe pump (NE-500 model, New Era Pump Systems, Inc., USA), and the flow rate was adjusted between 0.4 and 0.5 mL/h. In every case, the scaffolds were made from 1 mL of the solutions. The fibers were collected on an aluminum foil, which was placed on a slow rotary drum (10 rpm). The distance between the needle of the syringe and the collector was 15 cm in all cases. The applied voltages (direct current power supply, ES 30 Model, Gamma High Voltage Inc., USA) and flow rates are in Table 1.

### Fourier-transform infrared spectroscopy

The presence of inorganic salts in the scaffolds was investigated by using infrared spectroscopy. For this purpose, a JASCO FT/IR-4700 spectroscope with a diamond ATR head was applied. In all cases, the measurements were taken between 400 and 4000 cm^−1^ wave numbers with a 2 cm^−1^ resolution. A deuterated triglycine sulfate (DTGS) detector was used, and 256 scans were accumulated for each sample. The background spectra (H_2_O, CO_2_, ATR head exclusion) were obtained on a diamond crystal and were subtracted from the sample spectra. The peaks of the spectra were determined with the SpectraGryph software, and the graph was made using the Origin 2018 program (OriginLab, USA).

### Dissolution of the salts from the scaffolds

To prove the dissolution of inorganic salts from the scaffolds, the conductivity of the dissolution medium was measured. Stock solutions (50 mL, 5% w/v) were prepared from Sr(NO_3_)_2_ and Zn(O_2_CCH_3_)_2_ salts with distilled water. Then, by dilution, a concentration series was made for the calibration: 2.5, 1, 0.5, 0.2, 0.1, 0.05, and 0.01% (w/v). The conductivity of these solutions with different salt contents was determined using a conductivity meter and the belonging sensor (SevenCompact Duo S213 Benchtop pH/mV/Conductivity Meter; Cond Sensor InLab^®^ 710, Mettler Toledo, USA). The salt-containing scaffolds were wrapped into silk paper and dropped into 20 mL of distilled water to investigate how the salts can be dissolved from them. The solutions were stirred at 100 rpm such that the salt content could be properly distributed in distilled water. The samples for the measurements were taken from the media outside the wrapped scaffolds. The experiment was performed in 8 h: in the first hour conductivity values were measured every 10 min, then the measurements were taken every hour after that.

### Scanning electron microscopy and energy-dispersive X-ray spectroscopy

Scanning electron microscopy images were taken to determine the fiber diameters of salt-containing polymer scaffolds. They were made at the Budapest University of Technology and Economics, Department of Polymer Technology, applying a high-performance scanning electron microscope (JSM-6380LV, JEOL, Japan), with a resolution of 3.0 nm. Before the measurements, all the samples were fixed on a tape and coated with gold using a JFC-1200 Sputter Coating System (JEOL, Japan). Images of the samples were taken at 1000×, 5000×, and 10000× magnification, applying a 10 kV accelerating voltage. The fiber diameters of the scaffolds were determined by the Fiji ImageJ software (open-source software). In each case, 50 fiber diameters were measured. The results were statistically analyzed by the software GraphPad Prism 8.0.1 (GraphPad Inc., USA), including the normality tests regarding the distribution of fiber diameters (Shapiro–Wilk normality test) and the significant difference between different experimental groups (Kruskal–Wallis test). The fiber diameter distributions were plotted in the Origin 2018 program (OriginLab, USA). To complete the FTIR analysis, we used an energy-dispersive X-ray spectroscopy (EDX) detector during the SEM measurements to show the presence of salts in the fibers. The EDX spectra were taken at a voltage of 15 kV.

### Mechanical properties

A mechanical testing machine (4952, Instron, USA) and the associated software (Bluehill 2, USA) were used to examine the mechanical properties of the electrospun scaffolds. The tensile testing was conducted at room temperature. The cut samples were 6 cm long and 1.5 cm wide, and a 1 mm/min pulling speed was applied in each case. The specific load capacity (Equation 1) and the specific maximum load capacity (Equation 2) of each scaffold were determined, whose values were calculated according to the following equations:


[1] $$\text{Specific load capacity}\left(\frac{{\text{Nm}}^{2}}{\text{g}}\right)=\left(\frac{\text{Load (N)}}{\text{Area density}\left(\frac{\text{g}}{{\text{m}}^{2}}\right)}\right).$$

[2] $$\text{Specific max. load capacity}\left(\frac{{\text{Nm}}^{2}}{\text{g}}\right)=\frac{\text{Maximal load}\left(\text{N}\right)}{\text{Area density}\left(\frac{\text{g}}{{\text{m}}^{2}}\right)}.$$

From the elongation values and the length of the polymer scaffold captured at the beginning of the experiments, the percentage of elongation was determined using the following formula:


[3] $$\text{Elongation (\%) = }\frac{\text{Extension (mm)}}{\text{Initial length (mm)}}\times 100.$$

Typical load–extension curves were available in the software belonging to the mechanical testing machine. These graphs were converted into specific load capacity/elongation curves by using Equation 1 and Equation 2. Data analysis was performed by using the specific maximum load capacity confidence intervals of the scaffolds.

The elongation at breakpoint values were determined from the elongation and load values. For statistical tests, a nonparametric one-way ANOVA analysis (Welch’s correction) was performed (GraphPad Inc., USA).

The stress–strain curves were also calculated from the load and extension values by using the following equations:


[4] $$\text{Stress (MPa)}=\frac{\text{Load (N)}}{{\text{Cross-sectional area (mm}}^{2}\text{)}}.$$

[5] $$\text{Strain = }\frac{\text{Extension (mm)}}{\text{Initial length (mm)}}.$$

The cross-sectional area of the scaffolds was calculated from the product of the thickness and the width. The thickness of the scaffolds was measured by a digital micrometer (IP65, Mitutoyo, Japan). The Young’s modulus was calculated from the linear part of the stress–strain curve [45]. The slope of the linear function gives the Young’s modulus.

### Antibacterial activity

The Kirby–Bauer disc diffusion method was applied to test the antibacterial activity of the scaffolds containing different salts [46–47]. The experiment was performed using four bacterial strains (American Type Culture Collection, ATCC): *E. coli* (Gram-negative), *P. aeruginosa* (Gram-negative), *S. epidermidis* (Gram-positive), and *B. subtilis* (Gram-positive). A 24 h culture was made from each strain, and an amount of inoculum was suspended into 5 mL of saline solution. The suspensions were diluted until their McFarland values reached 0.5, corresponding to 10^8^ colony-forming units (CFU)/mL; the measurement was determined by a DEN-1 densitometer (Biosan, Latvia). From the bacterial strains, bacterial lawns were made on the Muller–Hinton medium. The mass of previously sterilized discs (6 mm in diameter) made from salt-containing scaffolds was measured, and three parallel samples were placed on the medium. Then, 10 µL of DMSO was dropped onto one of them to examine whether the liquid agent changed the dissolution of the salts from the scaffolds. The bacterial cultures with the discs were incubated at 37 °C for 24 h, and then inhibition/diffuse zones were observed. The diameter of the inhibition zones is the area where bacteria do not appear at all. The diffuse zones defined in our paper are areas where the salt could diffuse out from the scaffold and start some clean-up zone in the agar (not a totally clean area from bacteria). These zones were measured by the ImageJ software, and with Equation 6, the specific antibacterial area was calculated [22]. The *d*_zone_ means the diameter of the inhibition or diffuse zone and *d*_scaffold_ means the diameter of the scaffolds after 24 h of incubation. The *m*_salt_ is the calculated mass of the salts in a scaffold disc (6 mm in diameter).


[6] $$\text{Specific antibacterial area }\left(\frac{{\text{cm}}^{2}}{\text{mg salt}}\right)=\frac{{\left(\frac{d_{\text{zone}}}{2}\right)}^{2}\cdot \pi -{\left(\frac{d_{\text{scaffold}}}{2}\right)}^{2}\cdot \pi }{m_{\text{salt}}}.$$

### In vitro cell studies

#### Cell cultures

An osteosarcoma cell line (MG-63, ECACC 86051601) and skin fibroblasts (155BR, ECACC 90011809), both of human origin, were cultured as monolayers at 37 °C and 5% CO_2_ in a humidified atmosphere, and subcultivated at a subconfluent state by using trypsin/EDTA (0.05%). The medium used for the MG-63 cell line was composed of MEM supplemented with 10% of fetal bovine serum, 1% of non-essential amino acids, 2 mM of ʟ-glutamine, 100 IU/mL of penicillin, and 100 µg/mL of streptomycin. The medium for the 155BR cells contained 15% of fetal bovine serum, 1% of non-essential amino acids, 2 mM of ʟ-glutamine, 100 IU/mL of penicillin, and 100 µg/mL of streptomycin in DMEM.

#### Cytotoxicity tests

Indirect cytotoxicity tests were based on a previous publications of our research group [34]. The tests were performed first on tumor cells to optimize the experimental conditions and then on fibroblasts to investigate if the scaffolds have a cytotoxic effect. The discs with diameters of 16 mm (*m* = 0.5 ± 0.1 mg) were sterilized under UV light for 1 h. After that, they were incubated in cell culture medium for 24 h (37 °C and 5% CO_2_). The mass of the discs varied, so the applied volume of culture medium was proportionally adjusted (0.1 mg of scaffold in 1 mL of medium). The control medium containing the same salt concentration as the scaffolds were sterile filtered by 0.2 µm syringe filters (Sarstedt AG & Co. KG, Germany).

Both cell types were seeded onto a 96-well plate at a concentration of 5000 cells/cm^2^. The names of the samples and the treatment of the cells are shown in Table 2. Eight parallel measurements were made for each sample.

**Table 2:** Names of the samples used in the cytotoxicity test and their treatments.

Sample name	Treatment

C	negative control, no treatment
DC	positive control, reportedly cytotoxic agent – 10% DMSO
PSI	extract from the 25 PSI scaffold
PSI_Sr5	extract from the 20 PSI + 5 Sr(NO_3_)_2_ scaffold
PSI_Sr7.5	extract from the 20 PSI + 7.5 Sr(NO_3_)_2_ scaffold
PSI_Sr10	extract from the 20 PSI + 10 Sr(NO_3_)_2_ scaffold
PSI_Zn5	extract from the 20 PSI + 5 Zn(O_2_CCH_3_)_2_ scaffold
Sr5	medium with the same Sr(NO_3_)_2_ content as the 20 PSI + 5 Sr(NO_3_)_2_ scaffold
Sr7.5	medium with the same Sr(NO_3_)_2_ content as the 20 PSI + 7.5 Sr(NO_3_)_2_ scaffold
Sr10	medium with the same Sr(NO_3_)_2_ content as the 20 PSI + 10 Sr(NO_3_)_2_ scaffold
Zn5	medium with the same Zn(O_2_CCH_3_)_2_ content as the 20 PSI + 5 Zn(O_2_CCH_3_)_2_ scaffold

After one day of incubation, the cells were investigated under a phase-contrast microscope (Nikon Eclipse TS100, Nikon, Japan), and photomicrographs were taken using a CCD camera (COHU, USA) to track any changes in cell morphology. Then, 100 µL of old medium was replaced in each well with 200 µL of media: positive or negative control, extracts from the scaffolds, or media with the same salt concentrations.

After 24 and 72 h treatments, phase-contrast microscopy images were taken. The relative cell viability was determined using the WST-1 reagent (diluted in medium without phenol red (colorless) at 1:20). A volume of 200 µL of old medium was removed from each well and 200 µL of phosphate-buffered saline was added to eliminate the residual media as well as cells that were either loosely attached or did not attach to the plate. Then, 100 µL of diluted WST-1 reagent was added to the cells and incubated for 4 h. The absorbance values of the samples were measured in a microplate reader (model 3550, Bio-Rad Laboratories, Japan) at 450 nm. A reference wavelength of 655 nm was also used. The statistical analysis of the relative cell viability values was done using one-way ANOVA in the GraphPad Prism 8.0.1 software (GraphPad Inc., USA).

## Results and Discussion

To create a proper wound dressing material containing inorganic salt for potential antibacterial purposes, the first step was the synthesis of the polymer, the characterization of the solution, and the final scaffold with different techniques.

### Characterization of solutions and scaffolds

**Figure 1:**
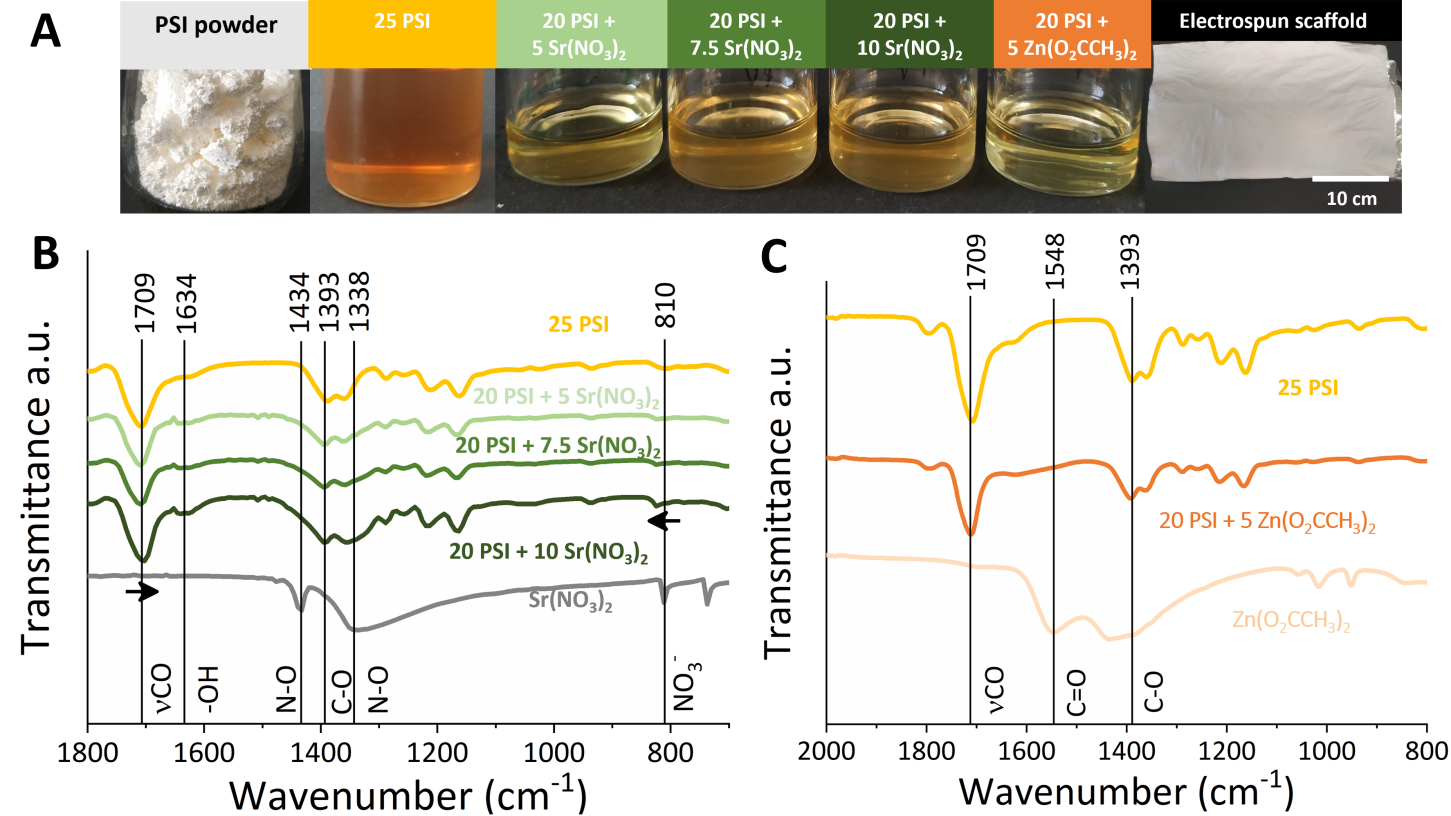
A) The synthesized PSI powder, 25 PSI solution, 20 PSI + salt solutions, and fibrous scaffolds were made using the electrospinning technique. B) FTIR spectra of the Sr(NO_3_)_2_ salt, scaffolds containing this salt, and 25 PSI scaffold. C) FTIR spectra of the Zn(O_2_CCH_3_)_2_ salt, scaffolds containing this salt, and 25 PSI scaffold.

The white PSI powder dissolved in DMF resulted in a deep-yellowish viscous solution (Figure 1A) as previously described [22,48]. By adding salts, the color deepened with the increased concentration of Sr(NO_3_)_2_, while a paler color appeared in the case of Zn(O_2_CCH_3_)_2_. These solutions were used for fiber formation using the electrospinning technique. All the created polymer nets were white in color, and the added salt did not cause macroscopic changes as we also experienced in our previous works [21,35]. During sample preparation, a small adjustment was made to the experimental conditions shown in Table 1. The presence of Sr(NO_3_)_2_ changed the viscosity and conductivity properties of the solutions. In the case of 20 PSI + 5 Sr(NO_3_)_2_ and 20 PSI + 7.5 Sr(NO_3_)_2_, a higher voltage was necessary for fiber preparation based on the viscosity decrease.

On the FTIR spectra (Figure 1B, Figure 1C), the main peaks of the PSI at 1709 cm^−1^ (νCO of –(OC)2N– asymmetric stretching vibration) and 1393 cm^−1^ (C–O bending vibration, δ) were perceptible [21,49]. FTIR images of the Sr(NO_3_)_2_ and 20 PSI + Sr(NO_3_)_2_ salts show that the characteristic peaks of this salt appeared in the salt-containing scaffold at 1434, 1338, and 810 cm^−1^. However, for the 810 cm^−1^ peak, there was a parallel shift towards higher wavenumbers after mixing with the polymer. This can indicate physical interaction between the polymer and the salt, although the chemical interaction would be a more characteristic change in the position of the peak. The 1338 and 1434 cm^−1^ peaks are attributed to the vibration of N–O, and the 810 cm^−1^ peak is accountable for the bending mode of NO^3^ [50]. At 1709 cm^−1^, the added Sr(NO_3_)_2_ salt caused a slight shift in the polymer peak. With the increasing salt concentration, the intensity of the peak increased, which was also observed by Sim and collaborators in the case of polymer electrolyte films containing LiCF_3_SO_3_ [51]. At 1634 cm^−1^, rising peaks also appeared as the concentration of salts increased. It was presumably the peak corresponding to bending vibration of water, H–O–H (Supporting Information File 1, Figure S1), which increased with a higher salt concentration since it could adsorb more water [52]. In the case of the 20 PSI + Zn(O_2_CCH_3_)_2_ scaffold, the peak of Zn(O_2_CCH_3_)_2_ appeared at 1548 cm^−1^, which is the symmetric peak and asymmetric stretch peak of the carbonyl group [53]. The PSI peaks did not change much after the addition of salts; therefore, it can be said that a physical mixture was formed between the polymer and the salts. The peaks associated with DMF did not appear either [54], indicating that the solvent completely evaporated during electrospinning. Although the presence of the salts was difficult to detect at this concentration, elemental analysis was performed with an EDX detector during the SEM experiments. In Supporting Information File 1, Figure S1, it can be seen that, in both cases, Zn and Sr appeared inside the fibers.

The presence of the salts was investigated using FTIR spectroscopy. However, for a wound dressing, the salts must leach out of the scaffolds to promote their local antibacterial effect on the desired environment. In distilled water, salt-containing scaffolds were soaked, and the conductivity of the solution was measured (Supporting Information File 1, Figure S2). At low salt concentrations, the conductivity in the aqueous solution is directly proportional to the concentration. Table 3 shows that for the samples containing Sr(NO_3_)_2_, almost 100% of the salinity was dissolved after 5–8 h. In the case of the Zn(O_2_CCH_3_)_2_ scaffold, only 48% of the maximal salt concentration was solubilized after 8 h, which is not in correlation with database information, since the solubility of Zn(O_2_CCH_3_)_2_ at 25 °C is 40 g/100 g of water [55]. This suggests that an interaction between PSI and Zn(O_2_CCH_3_)_2_ prevents the salt from entirely dissolving from the scaffold.

**Table 3:** The ratio of dissolution (in percentage) regarding Sr(NO_3_)_2_ and Zn(O_2_CCH_3_)_2_ salts from salt-containing PSI scaffolds after 1, 3, 5, and 8 h.

	1 h	3 h	5 h	8 h

20 PSI + 5 Sr(NO_3_)_2_	48%	58%	101%	106%
20 PSI + 7.5 Sr(NO_3_)_2_	47%	82%	101%	100%
20 PSI + 10 Sr(NO_3_)_2_	51%	77%	89%	98%
20 PSI + 5 Zn(O_2_CCH_3_)_2_	7%	27%	39%	48%

**Figure 2:**
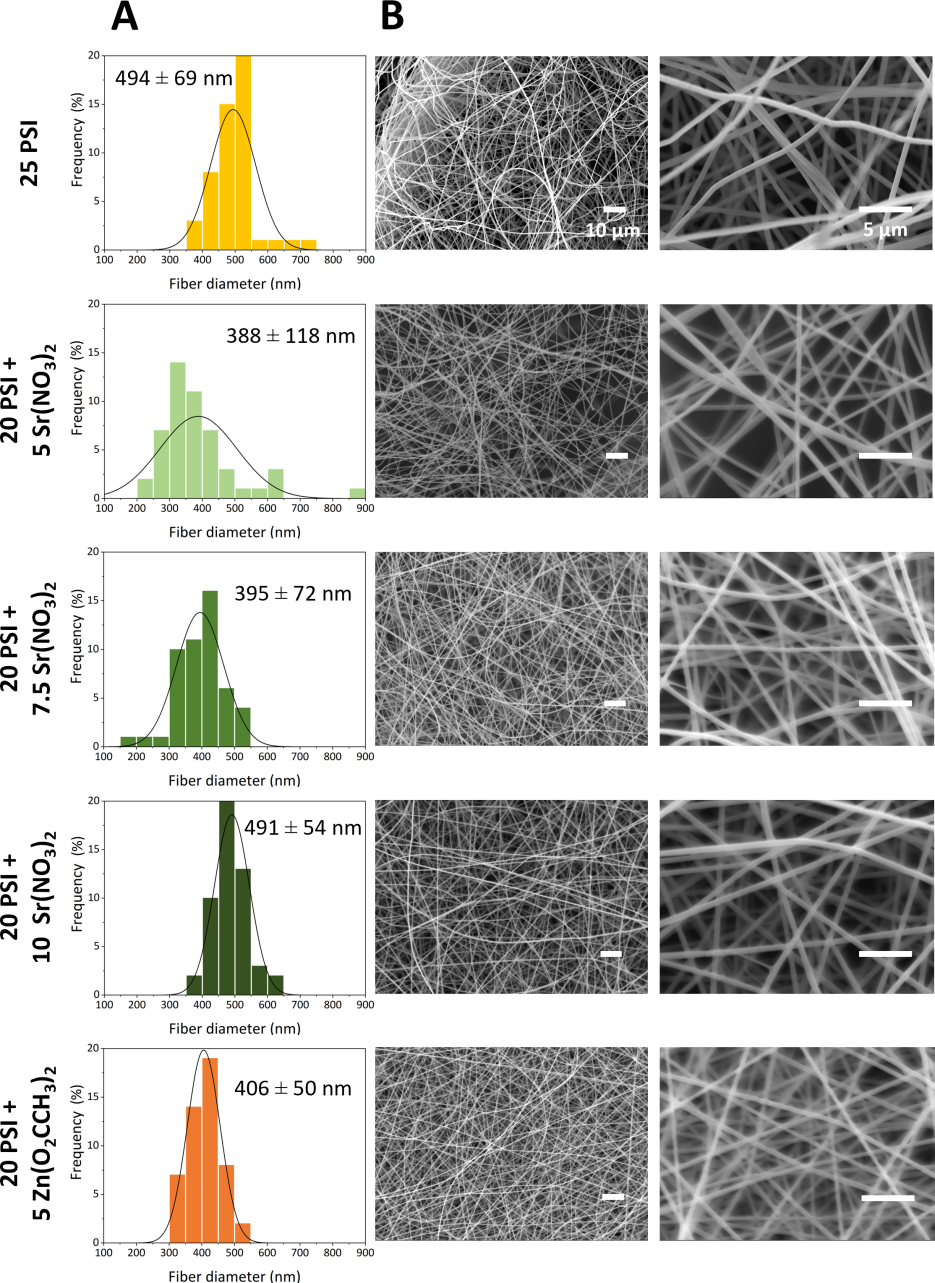
A) Fiber diameter distribution of PSI and PSI + salt scaffolds and B) SEM images at a lower (1000×) and a higher (5000×) magnification. The Gaussian lines on the histograms are guides to the eye.

In parallel with salt-dissolution measurements, a rigorous structural analysis was carried out based on the SEM images, and average fiber diameters were determined with a proper statistical analysis for each scaffold (Supporting Information File 1, Figure S3). Figure 2A shows the frequency of fiber diameters in percentage for each interval. The presence of Sr(NO_3_)_2_ and Zn(O_2_CCH_3_)_2_ salts in the fibers resulted in decreased diameter, which relates to increased conductivity and, in some cases, decreased viscosity. The salt-containing scaffolds include less polymers in % w/w, which could also cause lower average fiber diameter [56]. The fiber diameter values of 25 PSI and 20 PSI + 5 Sr(NO_3_)_2_ scaffolds do not follow a normal distribution (*p* < 0.05). However, 20 PSI + 7.5 Sr(NO_3_)_2_, 20 PSI + 10 Sr(NO_3_)_2_, and 20 PSI + 5 Zn(O_2_CCH_3_)_2_ can be described by a Gaussian distribution (*p* > 0.05). The Kruskal–Wallis statistical test was performed to investigate if the fiber diameter values or the shape of the fiber diameter distribution of the PSI and PSI + salt scaffolds are significantly different. Based on the results of the statistical analysis, 20 PSI + 5 Sr(NO_3_)_2_ (388 ± 118 nm), 20 PSI + 7.5 Sr(NO_3_)_2_ (395 ± 72 nm), and 20 PSI + 5 Zn(O_2_CCH_3_)_2_ (406 ± 50 nm) were significantly different from 25 PSI (494 ± 69 nm). However, in the case of the Sr(NO_3_)_2_ salt, the higher salt concentration increased the average fiber diameter and narrowed its distribution width. According to the statistical tests, in the case of salt-containing scaffolds, 20 PSI + 5 Sr(NO_3_)_2_ and 20 PSI + 7.5 Sr(NO_3_)_2_ were significantly different from the 20 PSI + 10 Sr(NO_3_)_2_ scaffolds. There was no significant difference between the 20 PSI + 5 Sr(NO_3_)_2_ and 20 PSI + 7.5 Sr(NO_3_)_2_ scaffolds. The two different salts in the 5% (w/w) concentration apparently showed a difference in the average fiber diameter and in the distribution of the fiber diameter. However, according to the statistical test, there was no significant difference between the 20 PSI + 5 Zn(O_2_CCH_3_)_2_ and the 20 PSI + 5 Sr(NO_3_)_2_ scaffolds. It is important to note that the Kruskal–Wallis test shows significant differences between groups when not only the medians and variances differ but also when the shape of the distribution is different. According to another survey where another polymer composite (polyacrylonitrile/polyvinylidene fluoride) containing different salts (NaCl, NaHCO_3_, CaCl_2_) was investigated, the presence of inorganic salts substantially decreased the average fiber diameter and made them more uniform [57]. Juhász et al. also investigated the effect of adding different salts in different concentrations to PSI with an outcome similar to our findings. In the case of CaCl_2_ and LiCl, it was observed that the increasing salt concentration caused increased fiber diameter and decreased distribution width. In the case of MgCl_2_, no perceptible trend concerning the aforementioned parameters was found by increasing the salt content [21]. Abdelhafiz et al. investigated polyvinylpyrrolidone (PVP) film doped with silver and zinc oxide nanoparticles. They reported that the presence of the nanoparticles increased the fiber diameter of the PVP polymer film [47].

### Evaluation of mechanical tests

For wound dressing applications, mechanical properties, including strength and elasticity of the fibrous scaffold, are crucial parameters. Medical gauze has a storage modulus of 1 MPa to 1 GPa [58], while the Young’s modulus of the skin is between 0.1 and 10 kPa, depending on the body parts, layer, and skin age [59]. In our research, we aimed to investigate the most critical mechanical parameters, such as the elongation at breakpoint, the specific maximum load capacity, and the Young’s modulus. Their values, presented in Table 4, describe the mechanical performance of different scaffolds.

**Table 4:** Mechanical parameters of the PSI and PSI + salt scaffolds.

Sample	Elongation at breakpoint (%)	Specific maximum load capacity (Nm^2^/g)	Young’s modulus (MPa)

25 PSI	47.8 ± 10.9	0.22 ± 0.04	35.4 ± 7.1
20 PSI + 5 Sr(NO_3_)_2_	14.9 ± 1.3	0.06 ± 0.01	170.1 ± 24.8
20 PSI + 7.5 Sr(NO_3_)_2_	7.7 ± 4.1	0.07 ± 0.01	373.2 ± 58.2
20 PSI + 10 Sr(NO_3_)_2_	7.0 ± 0.7	0.09 ± 0.01	143.3 ± 7.7
20 PSI + 5 Zn(O_2_CCH_3_)_2_	37.6 ± 5.9	0.05 ± 0.02	12.2 ± 1.2

**Figure 3:**
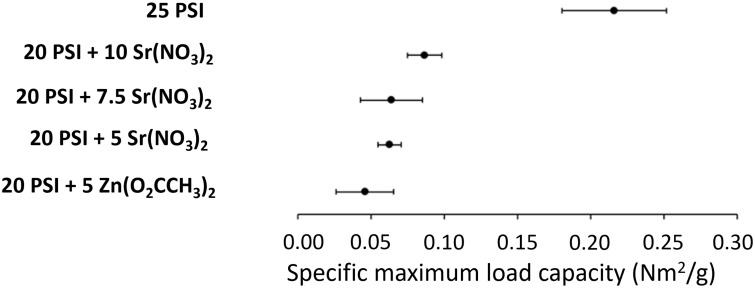
The specific maximum load capacity confidence interval of PSI and PSI + salt scaffolds.

The specific load capacity values of the scaffolds were determined according to the specific load capacity–elongation curves (Supporting Information File 1, Figure S4). The maximum load capacity is calculated from the maximal force that the mechanical instrument can exert on the test material divided by the specific weight. The calculated specific maximum load capacity values of the scaffolds were plotted with their confidence intervals (Figure 3). Adding salts to the polymer changed the shape of the curve of the PSI, which can be explained by the physical interaction between the polymer and the salts (Supporting Information File 1, Figure S4). The value of the salt-containing scaffold was reduced compared to that of the 25 PSI scaffold, whose specific maximum load capacity value was 0.22 ± 0.04 N·m^2^/g. The salt content was expected to improve the mechanical properties [60]. The specific maximum load capacity values were overlapped for the fibrous samples 20 PSI + 7.5 Sr(NO_3_)_2_ (0.07 ± 0.01 N·m^2^/g) and 20 PSI + 10 Sr(NO_3_)_2_ (0.09 ± 0.01 N·m^2^/g). The value for the 20 PSI + 5 Sr(NO_3_)_2_ (0.06 ± 0.01 N·m^2^/g) scaffold was reduced compared to those with higher Sr(NO_3_)_2_ content. This value was even lower for the Zn(O_2_CCH_3_)_2_-containing scaffold (0.05 ± 0.02 N·m^2^/g). Similarly, Tóth et al. saw that drug loading decreased the specific maximum load capacity of the PSI polymer [35]. Pázmány et al. also investigated a scaffold with 25% (w/w) PSI content, and they measured a lower specific maximum load capacity (0.08 ± 0.01 N·m^2^/g) compared to that of our results at the same concentration but at a higher average fiber diameter (590 ± 124 nm) [34]. These results are in good agreement with the literature; the thinner the fiber, the higher the tensile strength [61–62].

The Young’s modulus of the scaffolds was calculated from the slopes of the linear parts of the stress–strain curves (Supporting Information File 1, Figure S5). In the case of the Sr(NO_3_)_2_ salt, the Young’s modulus was maximum at 7.5% (w/w, Table 4). The Zn(O_2_CCH_3_)_2_ salt (12.2 ± 1.2 MPa) caused a major reduction in the Young’s modulus value compared to that of Sr(NO_3_)_2_ salt-containing scaffolds or the pure scaffold. Although in the case of the 25 PSI scaffold this value was also very low (35.4 ± 7.1 MPa), this is not correlated with the specific maximum load capacity and the elongation at breakpoint values. Tóth et al. investigated the initial modulus of drug-loaded PSI scaffolds instead of Young’s modulus since the thickness of the scaffolds was hard to define. According to their results, a growing concentration of prednisone in PSI and PCL polymers also increased the value of the initial modulus. In the case of poly(vinyl alcohol) (PVA), the initial modulus values decreased with increasing prednisone content [35]. We also observed that the presence of strontium salt significantly increased the Young’s modulus. Nevertheless, the reason of this effect of adding strontium still needs to be clarified. Our hypothesis is that the presence of the salt increases the crystallinity of the fibers, which indicates that the elastic deformation phase is shortened and the Young’s modulus is increased. Therefore, the fibrous samples become more fragile.

**Figure 4:**
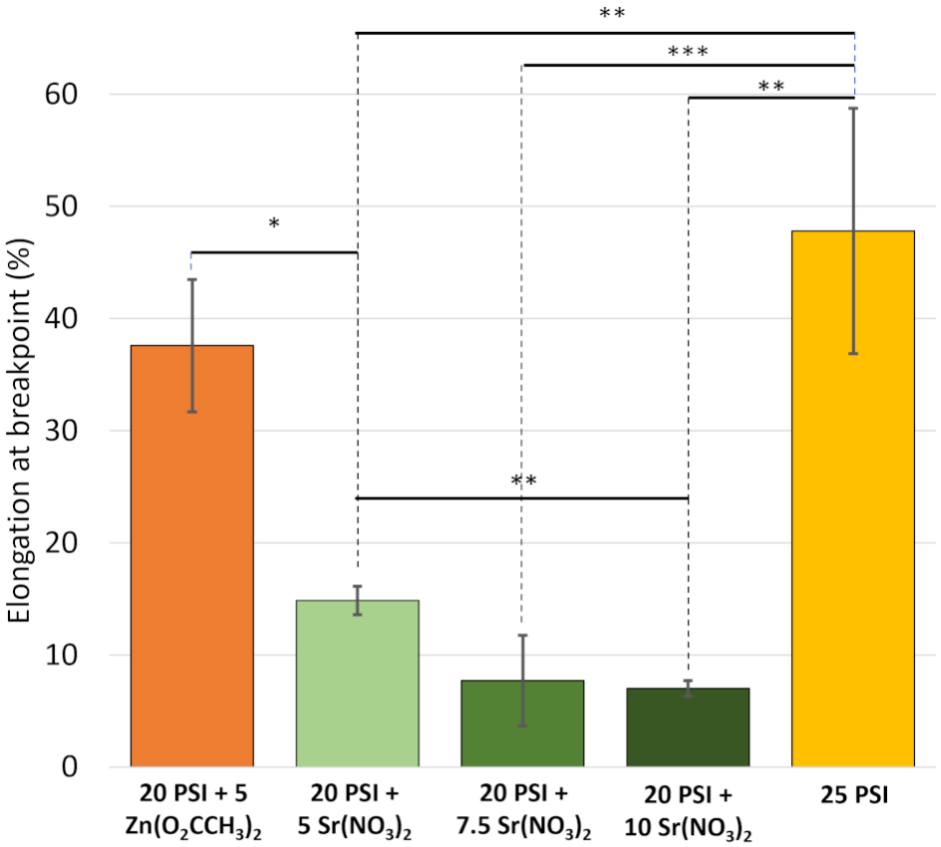
The elongation at breakpoint values of PSI and PSI + salt scaffolds. * indicates the significant difference between the two groups (Welch’s correction, *p < 0.05; **p < 0.01; ***p < 0.001).

The elongation at breakpoint values (Figure 4 and Supporting Information File 1, Figure S4) show the rigidity of the scaffolds. The Sr(NO_3_)_2_ salt-containing samples substantially reduced this parameter compared to that of other scaffolds which contained only the polymer or another salt. According to statistical tests, the elongation at breakpoint of the 25 PSI (47.8 ± 10.9%) scaffold was significantly different from that of 20 PSI + 5 Sr(NO_3_)_2_ (14.9 ± 1.3%), 20 PSI + 7.5 Sr(NO_3_)_2_ (7.7 ± 4%), and 20 PSI + 10 Sr(NO_3_)_2_ (7 ± 0.7%) samples. The two different salts caused a distinct effect on elongation at breakpoint values. The Zn(O_2_CCH_3_)_2_ did not significantly decrease the elasticity of the scaffold compared to that of the 25 PSI scaffold. The elasticity of 20 PSI + 5 Zn(O_2_CCH_3_)_2_ (37.6 ± 5.9%) was significantly different from the 20 PSI + 5 Sr(NO_3_)_2_ scaffold, even though they had the same salt content. In this respect, there was also a significant difference between the 20 PSI + 5 Sr(NO_3_)_2_ fibrous scaffold and the 20 PSI + 10 Sr(NO_3_)_2_ sample, but no difference was observed between 5 and 7.5% (w/w) Sr(NO_3_)_2_ and 7.5 and 10% (w/w) Sr(NO_3_)_2_ salt-containing scaffolds. The increasing amount of the Sr(NO_3_)_2_ salt made the scaffolds less elastic. Tóth et al. observed that after loading prednisone into PSI and PVA, the maximum elongation of the polymer significantly decreased. However, in the case of PCL, they found that prednisone increased the maximum elongation of the polymer [35]. Pázmány et al. reported that the elongation at breakpoint value of the 25% (w/w) PSI was lower (16 ± 3%) compared to that of our results with the same concentration [34]. The difference in the average fiber diameter could also explain this. Another study investigated different inorganic salts (LiCl, MgCl_2_·6H_2_O, CaCl_2_, and AlCl_3_·6H_2_O) added to starch/PVA films. They perceived that the salts improved the mechanical properties, and the increasing amount of salts increased the elongation at break values, which could reach up to 565.99% [63].

### Antibacterial activity of salt-containing scaffolds

Suppose we apply a scaffold that contains an antibacterial agent (in our case, salt) for wound dressing. It is important to note that it would not just be a mechanical barrier from the pathogens but also an active protection.

Only a few studies have demonstrated the antibacterial effects of zinc against *S. mutans* [64] and of strontium against *S. aureus* and *E. coli* [65]. Our work determined the inhibition and diffuse zones of four bacterial strains (*E. coli*, *P. aeruginosa*, *S. epidermidis*, and *B. subtilis)*. The bacterial strains *S. epidermidis*, *B. subtilis*, and *E. coli* are relevant because they could also be found in the human body, and they can be pathogenic to humans [66–68]. *P. aeruginosa* is also a pathogen that causes the most frequent nosocomial infections [69]. In most cases, it was observed that the scaffolds (6 mm in diameter) shrank during the incubation period on the agar gel, which was also observed in the case of electrospun scaffolds under certain conditions [70]. The specific antibacterial areas were defined (Table 5) by the diameter of the fibrous samples and the inhibition/diffuse zones after the 24 h-long experiments (Supporting Information File 1, Figure S6) using Equation 6.

**Table 5:** The antibacterial activity of different PSI + salt scaffolds against *B. subtilis* and *S. epidermidis* (Gram-positive), and *E. coli* and *P. aeruginosa* (Gram-negative) strains. IZ – inhibition zone; DZ – diffuse zone; SAA – specific antibacterial area; e – equal to IZ; n.d. – no data.

	*B. subtilis*					*S. epidermidis*			
		
	IZ (mm)	DZ (mm)	SAA (cm^2^/mg salt)			IZ (mm)	DZ (mm)	SAA (cm^2^/mg salt)	
		
			IZ	DZ				IZ	DZ
		
20 PSI + 5 Sr(NO_3_)_2_	5.2 ± 0.5	0.0	6.1	e		5.9 ± 1.0	10.0 ± 1.1	0.0	51.1
20 PSI + 7.5 Sr(NO_3_)_2_	5.6 ± 0.2	0.0	7.2	e		5.9 ± 0.0	0.0	12.1	e
20 PSI + 10 Sr(NO_3_)_2_	5.5 ± 0.1	0.0	2.7	e		5.5 ± 0.5	0.0	n.d.	e
20 PSI + 5 Zn(O_2_CCH_3_)_2_	5.7 ± 0.4	14.2 ± 1.6	n.d.	79.1		5.9 ± 0.7	8.0 ± 0.9	n.d.	13.5
									
	*E. coli*					*P. aeruginosa*			
		
	IZ (mm)	DZ (mm)	SAA (cm^2^/mg salt)			IZ (mm)	DZ (mm)	SAA (cm^2^/mg salt)	
		
			IZ	DZ				IZ	DZ
		
20 PSI + 5 Sr(NO_3_)_2_	6.1 ± 0.4	0.0	19.8	e		5.4 ± 0.5	0.0	14.0	e
20 PSI + 7.5 Sr(NO_3_)_2_	5.7 ± 0.9	9.4 ± 2.7	10.6	18.2		6.0 ± 0.3	0.0	12.3	e
20 PSI + 10 Sr(NO_3_)_2_	5.5 ± 0.6	0.0	5.1	e		5.4 ± 0.7	0.0	4.2	e
20 PSI + 5 Zn(O_2_CCH_3_)_2_	6.2 ± 1.3	0.0	9.4	e		4.7 ± 0.6	0.0	n.d.	e

The 20 PSI + 5 Sr(NO_3_)_2_ sample showed the highest inhibition capacity against *E. coli* (19.8 cm^2^/mg) based on the specific antibacterial area calculated by the inhibition zone. This sample also showed inhibition against *B. subtilis* (6.1 cm^2^/mg) and *P. aeruginosa* (14 cm^2^/mg) strains. A diffuse zone evolved on the *S. epidermidis* lawn (51.1 cm^2^/mg). The 20 PSI + 7.5 Sr(NO_3_)_2_ sample caused inhibition against *P. aeruginosa* (12.3 cm^2^/mg), *S. epidermidis* (12.1 cm^2^/mg), *E. coli* (10.6 cm^2^/mg), and *B. subtilis* (7.2 cm^2^/mg) strains. A diffuse zone appeared on the *E. coli* (18.2 cm^2^/mg) lawn. The 20 PSI + 10 Sr(NO_3_)_2_ sample showed low inhibition against *E.coli* (5.1 cm^2^/mg), *P. aeruginosa* (4.2 cm^2^/mg), and *B. subtilis* (2.7 cm^2^/mg). The 20 PSI + 5 Zn(O_2_CCH_3_)_2_ scaffold caused inhibition against the *E. coli* (9.4 cm^2^/mg) strain but not against the other strains. Diffuse zones appeared around the samples on the lawn of *B. subtilis* (79.1 cm^2^/mg) and *S. epidermidis* (13.5 cm^2^/mg). The presence of DMSO did not significantly change the inhibition zones; however, it caused the dissolution of the scaffolds.

Previously, Barczikai et al. investigated the effects of PSI scaffold against bacterial strains, and they noticed that narrow, clear zones were formed after 24 h of incubation. This phenomenon was explained by the change in pH during the hydrolyzation of the PSI. By adding silver nanoparticles to the PSI scaffold, the specific antibacterial areas were more extensive (20–60 cm^2^/mg) than we experienced for the same bacteria strains in the case of Sr(NO_3_)_2_ and Zn(O_2_CCH_3_)_2_ salt-containing scaffolds. These values were even higher when 5 mg/mL of paracetamol was added to them [22]. In another study, electrospun gelatin/Cs–Si (chitosan–silicone hybrid) fibers were made with zinc additives. They found that the scaffold had antibacterial activity against *S. aureus*, *B. subtillis*, *E. coli*, and *P. aeruginosa* bacterial strains [71]. Colinas et al. examined Zn-based coordination polymers in broth dilution and agar diffusion tests, and they saw effective antibacterial activity against both *E. coli* and *S. epidermidis* [30]. Almoudi et al. used 0.32 mg of Zn acetate/disc and had 12 and 18 mm inhibition zones for *S. mutans* and *S. sobrinus,* respectively. We applied Zn acetate in a concentration that was one order of magnitude lower (0.017 mg/disc), and observed 14.2 ± 1.6 mm and 8 ± 0.9 mm diffusion zones for *B. subtilis* and *S. epidermidis*, respectively. In our case, clear inhibition zones were not formed, and only diffuse zones appeared due to low salt concentration [72].

### Indirect cytotoxicity of salt-containing scaffolds

**Figure 5:**
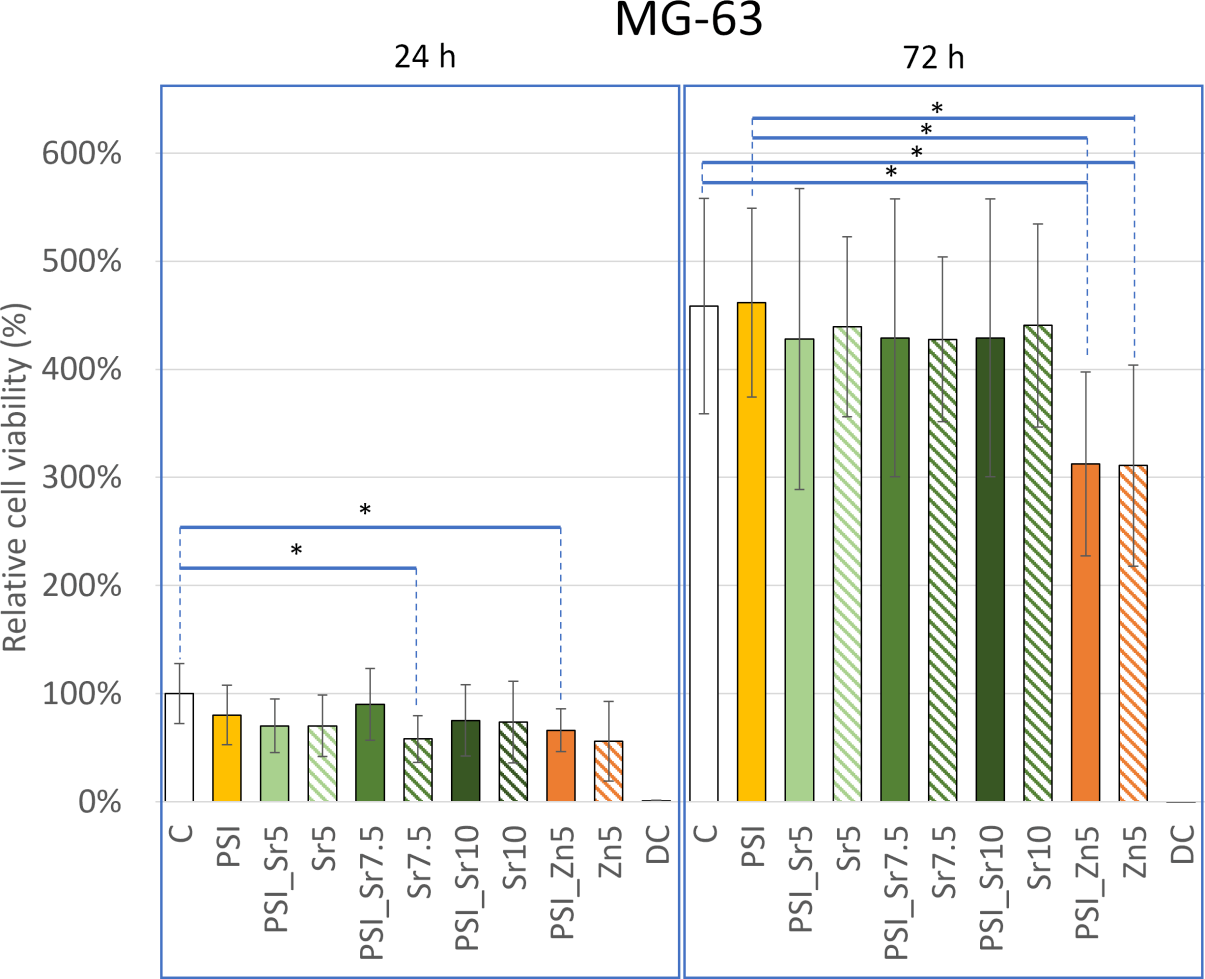
Relative cell viability of MG-63 tumor cells after 24 and 72 h compared to that of the 24 h control cells. * indicates significant differences (one-way ANOVA, *p < 0.05).

Our first cytotoxicity assays were performed on the MG-63 tumor cell line derived from the cells of a 14-year-old boy with osteosarcoma [73]. According to the ISO 10993-5:2009 standard, there are various levels of cytotoxicity. If there is no significant reduction in cell count compared to that of the negative control, then the substance is not cytotoxic. If more than 80% of the treated cells are viable compared to the untreated control cells, the substance is considered slightly cytotoxic; if the viability is between 50 and 80%, the substance is mildly cytotoxic; if the viability is below 50%, the substance is moderately cytotoxic; and if the viability is close to 0% then the substance is severely cytotoxic. For the determination of the extract, we used a new sample nomenclature (Table 2). After 24 and 72 h treatments, the relative cell viability was compared to that of the 24 h negative control (Figure 5). After 24 h, the morphology of the cells was unchanged in the case of each sample compared to the control cells based on phase-contrast microscopy images (Supporting Information File 1, Figure S7). The statistical results showed that after 24 h, there was no significant difference between the effect of each medium containing only salt (Sr5: 70 ± 29%; Sr7.5: 58 ± 22%; Sr10: 74 ± 38%; Zn5: 56 ± 37%) and the extracts of PSI + salt (PSI_Sr5: 70 ± 25%, PSI_Sr7.5: 90 ± 33%, PSI_Sr10: 75 ± 33%; PSI_Zn5: 66 ± 20%). No significant difference in viability was observed between the PSI extract (80 ± 27%) and the PSI + salt extract. The viability values for PSI_Zn5 and Sr7.5 samples were only significantly different from the 24 h negative control.

After 72 h, the cell number significantly increased, and the cells formed almost a confluent layer. There was a significant difference between the viability values of PSI_Zn5 (312 ± 85%) and Zn5 (311 ± 93%) samples compared to the 72 h negative control (458 ± 100%) and the PSI sample (462 ± 87%). Therefore, these Zn(O_2_CCH_3_)_2_-containing samples were mildly cytotoxic to MG-63 tumor cells. The cytotoxicity effects of other PSI + salt samples (PSI_Sr5: 428 ± 139%; PSI_Sr7.5: 429 ± 129%; PSI_Sr10: 441 ± 94%) were not significantly different from those of the PSI and negative control samples. Recently, Molnar et al. investigated the potential cytotoxicity of the hydrolyzed form of PSI, namely poly(aspartic acid) (PASP) on the MG-63 cell line. After 24 and 72 h, they observed no significant difference in the viability between the control (nontreated) and PASP-treated cells [74]. Furthermore, Juriga et al. found that MG-63 cells can proliferate on PASP-based hydrogels [75].

**Figure 6:**
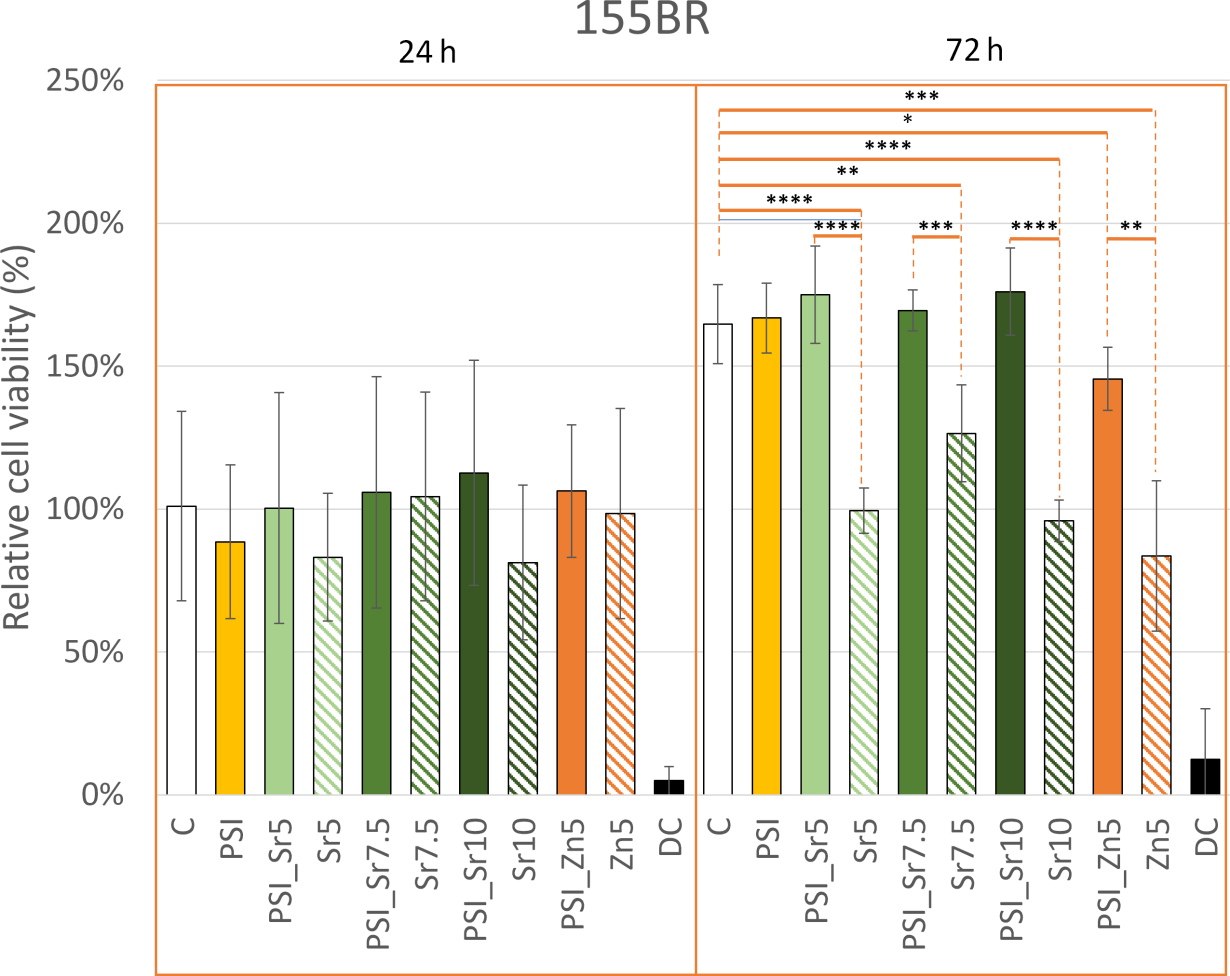
Relative cell viability of 155BR fibroblasts after 24 and 72 h compared to that of the 24 h control cells. * indicates significant differences (one-way ANOVA, *p < 0.05; **p < 0.01; ***p < 0.001; ****p < 0.0001).

Like the tumor cells, 155BR human fibroblasts were also subjected to cytotoxicity assays (Figure 6). After 24 h, there was no change in the morphology of treated cells compared to that of control cells based on phase-contrast microscopy images (Supporting Information File 1, Figure S8). According to the statistical tests, there was no significant difference between the viability after treating the cells with the media containing only salts (Sr5: 83 ± 22%; Sr7.5: 104 ± 37%; Sr10: 81 ± 27%; Zn5: 98 ± 37%) and the extract from PSI + salt scaffolds (PSI_Sr5: 100 ± 40%; PSI_Sr7.5: 106 ± 40%; PSI_Sr10: 113 ± 39%; PSI_Zn5: 106 ± 23%). We found no significant difference between the viability of the 24 h negative control and the salt, PSI, or PSI + salt-treated cells. After 72 h, the viability of the cells increased by a little over 1.5 times compared to that of the 24 h values. This growth rate was slightly lower than expected since the duplication time of 155BR cells is approximately 32 h [76]. The PSI (167 ± 12%), PSI_Sr5 (175 ± 17%), PSI_Sr7.5 (169 ± 7%), and PSI_Sr10 (176 ± 15%) samples were not significantly different from the 72 h negative control samples (165 ± 14%). The viability of salt-containing samples (Sr5: 99 ± 8%; Sr7.5: 126 ± 17%; Sr10: 96 ± 7%; Zn5: 84 ± 26%) and PSI_Zn5 (146 ± 11%) were significantly different from that of the 72 h negative control. As can be seen on the phase-contrast microscopy images, the cell number was higher in the case of the treatment with the extract from the PSI + salt scaffolds than that in the case of treatment with media containing only salts. The statistical test confirmed this observation, showing significantly higher viability values for PSI + salt samples compared to that of the same salt alone. Based on the average relative cell viability, the PSI_Zn5 sample was slightly cytotoxic, while the Sr5, Sr7.5, Sr10, and Zn5 samples were mildly cytotoxic to 155BR fibroblasts. Pázmány et al. also demonstrated that the extract of the hydrolyzed form of PSI cross-linked with 1,4-diaminobutane is not toxic to 155BR fibroblasts after 24 and 72 h of incubation [34].

Dodero et al. investigated the cytotoxicity of the extract from alginate membranes cross-linked with Sr^2+^ ions. They reported no sign of cytotoxicity on L929 mouse fibroblasts and HaCaT human keratinocytes after 24 and 48 h of incubation [31].

## Conclusion

Based on FTIR spectroscopy, both salts formed a physical mixture with polysuccinimide. In the case of Sr(NO_3_)_2_, 100% of dissolution was observed after 5–8 h, while in the case of Zn(O_2_CCH_3_)_2_, approximately 50% of the salt content was dissolved from the scaffold. The polymer scaffolds containing Sr(NO_3_)_2_ or Zn(O_2_CCH_3_)_2_ showed lower fiber diameters than those of the PSI scaffold. Adding salts to the polymer also caused changes in the mechanical properties of the scaffold: Sr(NO_3_)_2_ made the scaffolds more rigid, but it remarkably increased the Young’s modulus, although the Zn(O_2_CCH_3_)_2_ did not significantly change the elongation at breakpoint. The specific maximum load capacity values also decreased each time after adding salts to the PSI. The antibacterial experiments showed that the Zn(O_2_CCH_3_)_2_-containing scaffold was effective against *B. subtilis*, and the Sr(NO_3_)_2_-containing scaffold eliminated *E. coli* and *P. aeruginosa*. The cytotoxicity tests indicated that Sr(NO_3_)_2_-containing scaffolds were not cytotoxic, and the 20 PSI + 5 Zn(O_2_CCH_3_)_2_ scaffold was slightly cytotoxic to the 155BR fibroblasts after 72 hours. Based on previous data and on our current findings, we are confident that the PSI scaffolds with appropriate concentrations of Sr(NO_3_)_2_ and Zn(O_2_CCH_3_)_2_ could serve as potential wound dressings to perform local antibacterial release from nanoscale fibrous biocompatible scaffolds in an environmentally friendly way by avoiding the potential risk of AMR.

## Supporting Information

The Supporting Information file contains additional FTIR spectra (1000–1800 cm^−1^) of water and full spectra (4000–500 cm^−1^) of strontium nitrate, zinc acetate salts, 25 PSI scaffold, and 20 PSI + salt scaffolds; results of the dissolution test; the process of the determination of the fiber diameters; elongation-specific load capacity graphs of different scaffolds; stress–strain curves and the process of the determination of the Young’s modulus of the scaffolds; results of the antibacterial activity test; and phase-contrast microscopy images of MG-63 and 155BR cells during cytotoxicity tests.

File 1 Additional experimental data.

## Data Availability

All data that supports the findings of this study is available in the published article and/or the supporting information to this article.
