# Supplementary material for: Electrospun polysuccinimide scaffolds containing different salts as potential wound dressing material
Source: Beilstein J Nanotechnol. 2024 Jul 2;15:781–96. doi: 10.3762/bjnano.15.65 (PMC11228618; doi:10.3762/bjnano.15.65)
Supplement: File 1 — Additional experimental data. [file Beilstein_J_Nanotechnol-15-781-s001.pdf]

BEILSTEIN JOURNAL OF NANOTECHNOLOGY

Supporting Information

for

Electrospun polysuccinimide scaffolds containing different salts as potential wound dressing material

Veronika Pálos, Krisztina S. Nagy, Rita Pázmány, Krisztina Juriga-Tóth, Bálint Budavári, Judit Domokos, Dóra Szabó, Ákos Zsembery and Angela Jedlovszky-Hajdu

Beilstein J. Nanotechnol. **2024,** *15,* 781–796. doi:10.3762/bjnano.15.65

Additional experimental data

License and Terms: This is a supporting information file under the terms of the Creative Commons Attribution License (https://creativecommons.org/licenses/by/4.0). Please note that the reuse, redistribution and reproduction in particular requires that the author(s) and source are credited and that individual graphics may be subject to special legal provisions.
The license is subject to the *Beilstein Journal of Nanotechnology* terms and conditions: (https://www.beilstein-journals.org/bjnano/terms)

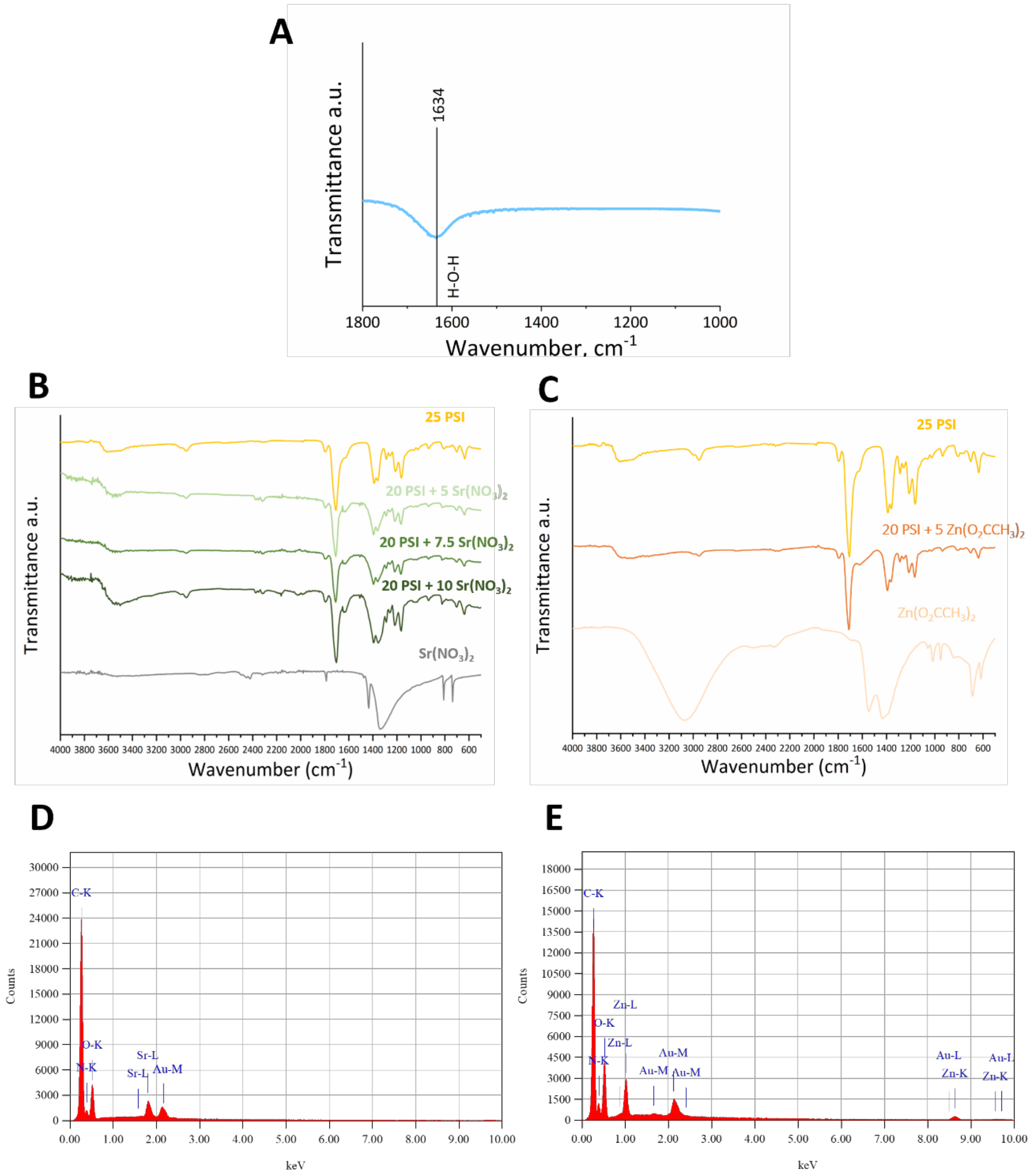

Figure S1: A) The FTIR spectra of water with the characteristic H–O–H bending vibration peak at 1634 cm^{-1}. B) FTIR spectra of the $Sr(NO_3)_2$ salt, scaffolds containing this salt, and 25 PSI scaffold between 4000 and 500 cm^{-1}. C) FTIR spectra of the $Zn(O_2CCH_3)_2$ salt, scaffold containing this salt, and 25 PSI scaffold between 4000 and 500 cm^{-1}. D) EDX spectrum of the 20 PSI + 5 $Sr(NO_3)_2$ scaffold E) EDX spectrum of the 20 PSI + 5 $Zn(O_2CCH_3)_2$ scaffold.

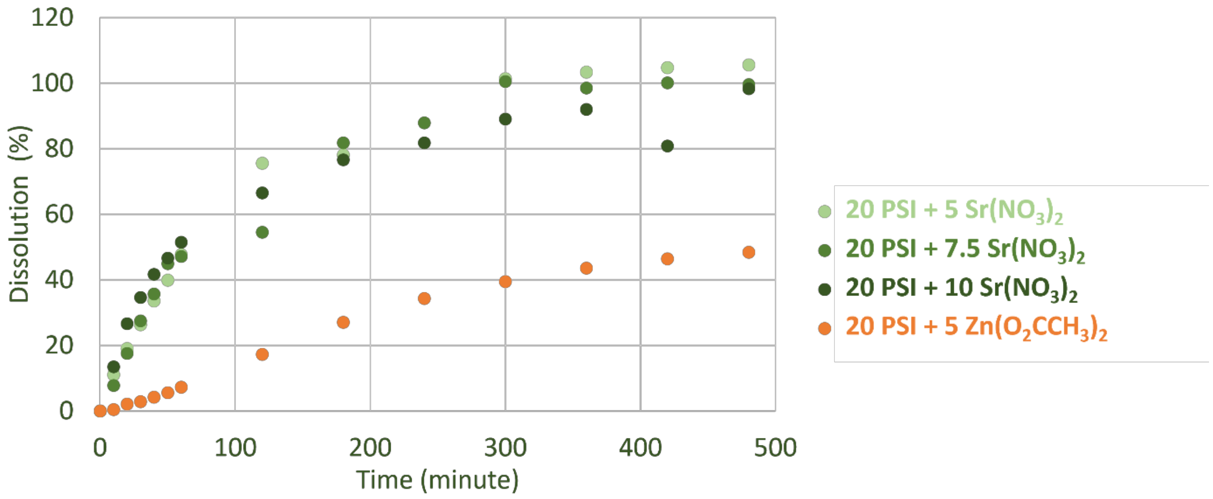

Figure S2: The dissolution of the scaffolds with different salt content in distilled water for 8 h.

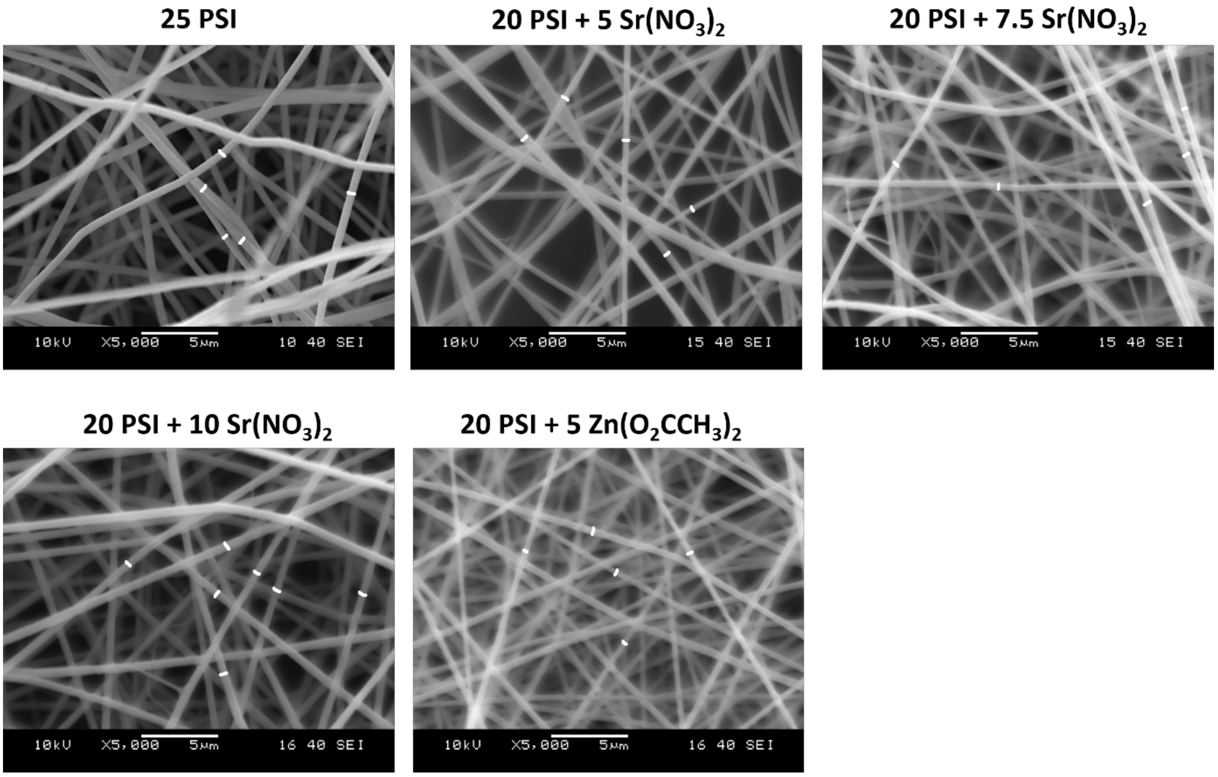

Figure S3: The process of fiber diameter determination by ImageJ.

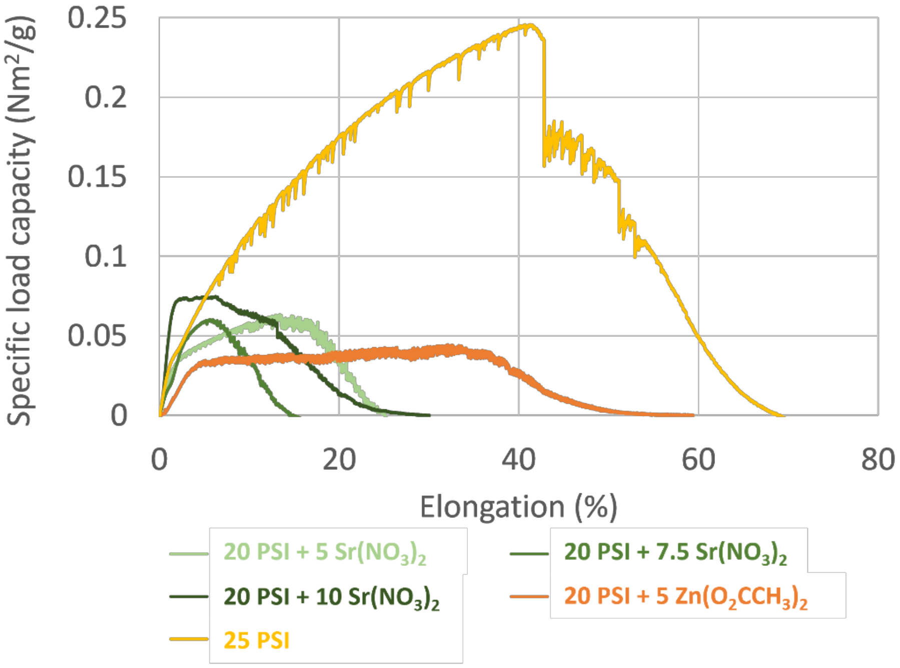

Figure S4: Elongation-specific load capacity graphs of different scaffolds.

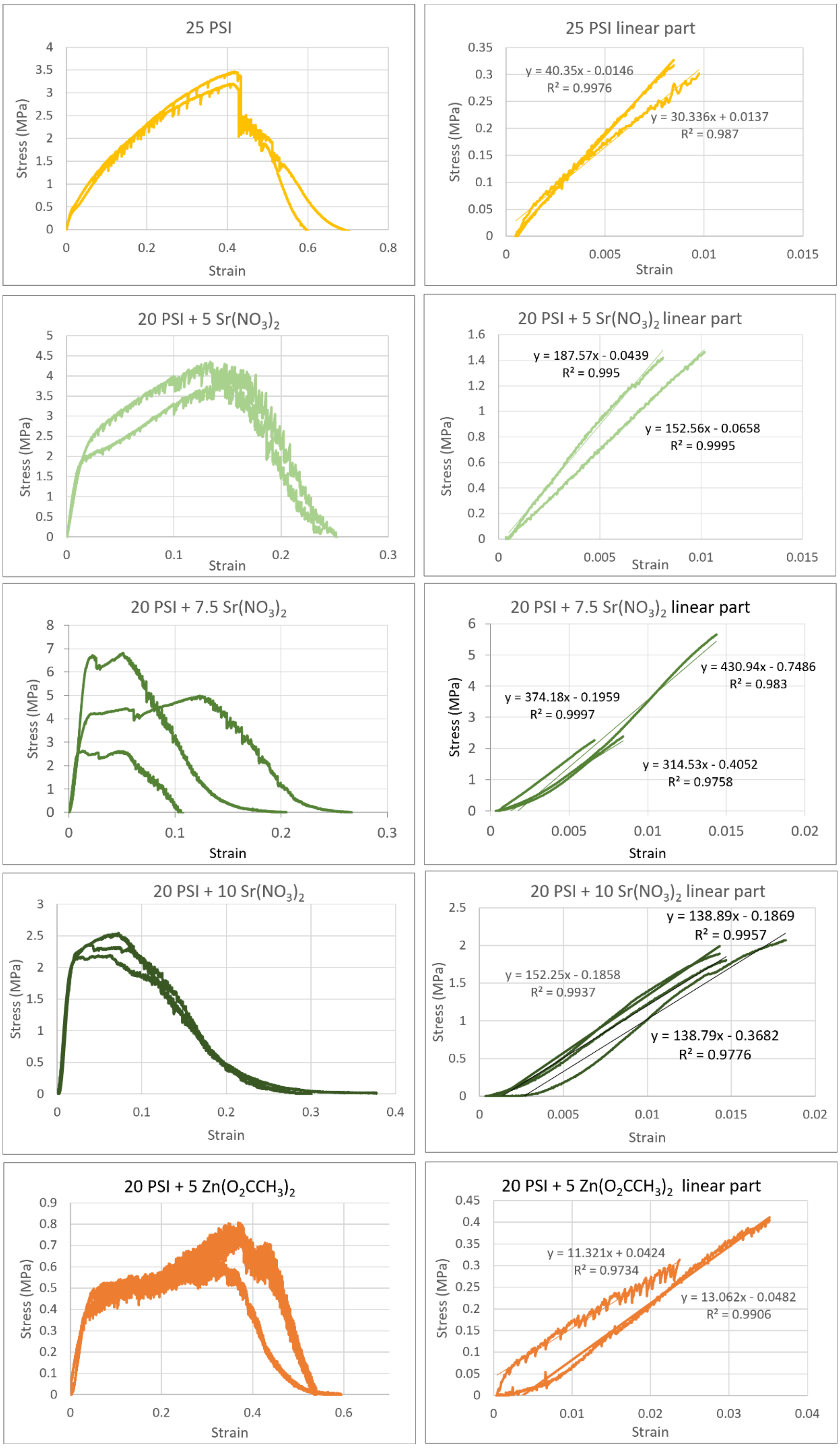

Figure S5: Typical stress–strain curves of 25 PSI and PSI + salt scaffolds, and the determination of the Young's modulus from the linear part of the curve.

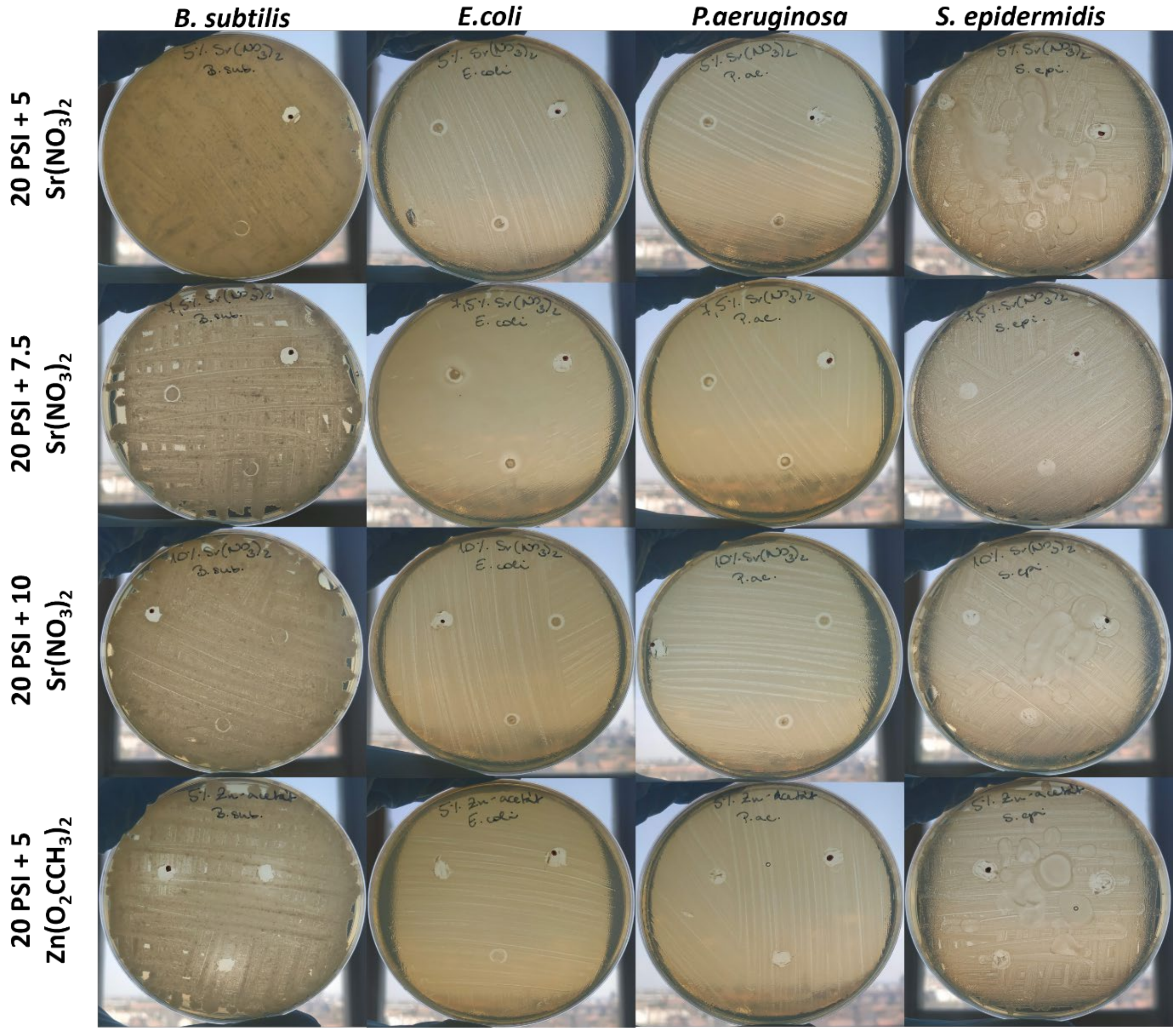

Figure S6: The inhibition and diffuse zones of salt-containing scaffolds on four different bacteria lawns.

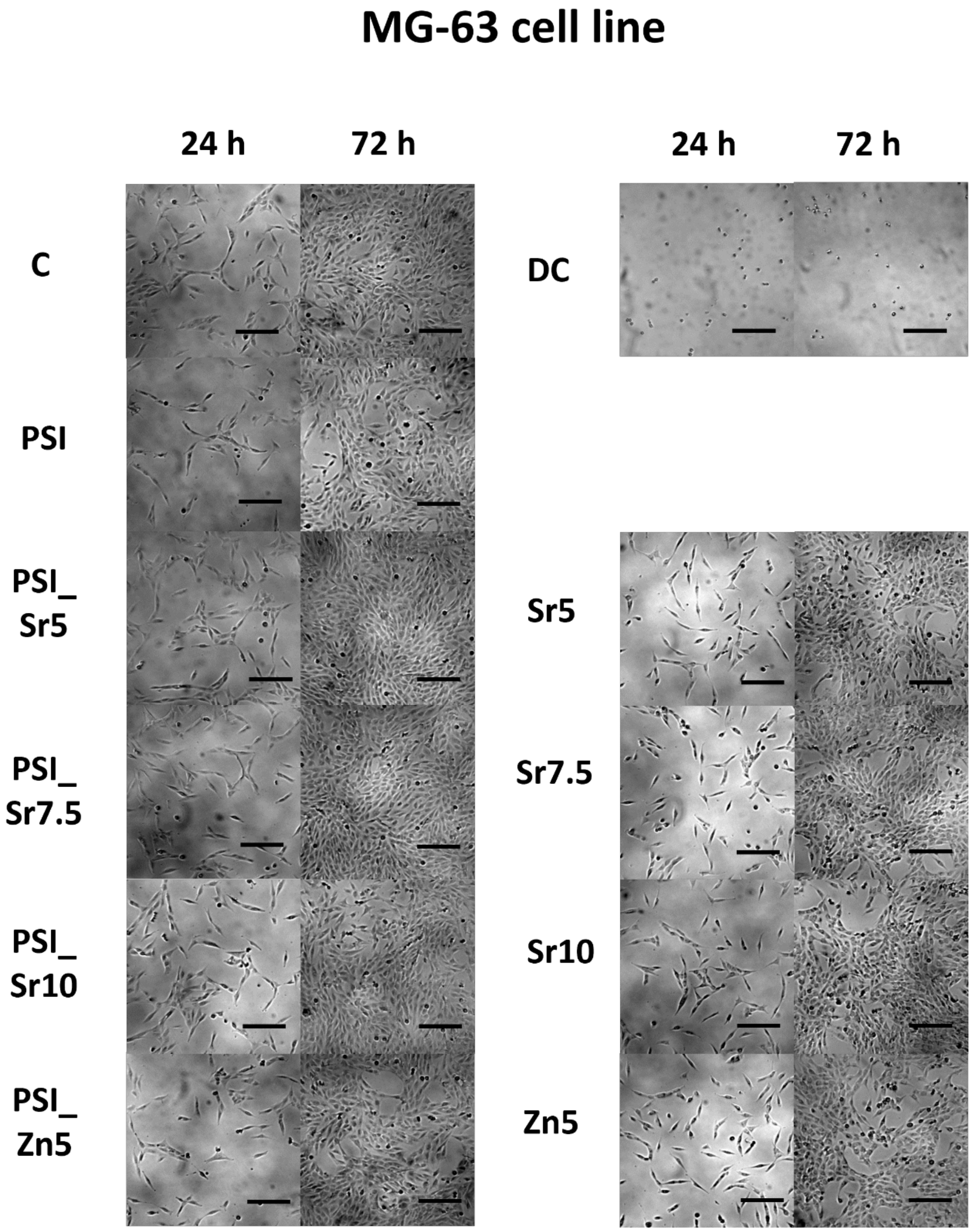

Figure S7: Phase-contrast microscopy images of the MG-63 cell line after treatment for 24 and 72 h with different concentrations of salts and extract of the scaffolds. The scale bars indicate 100 μm.

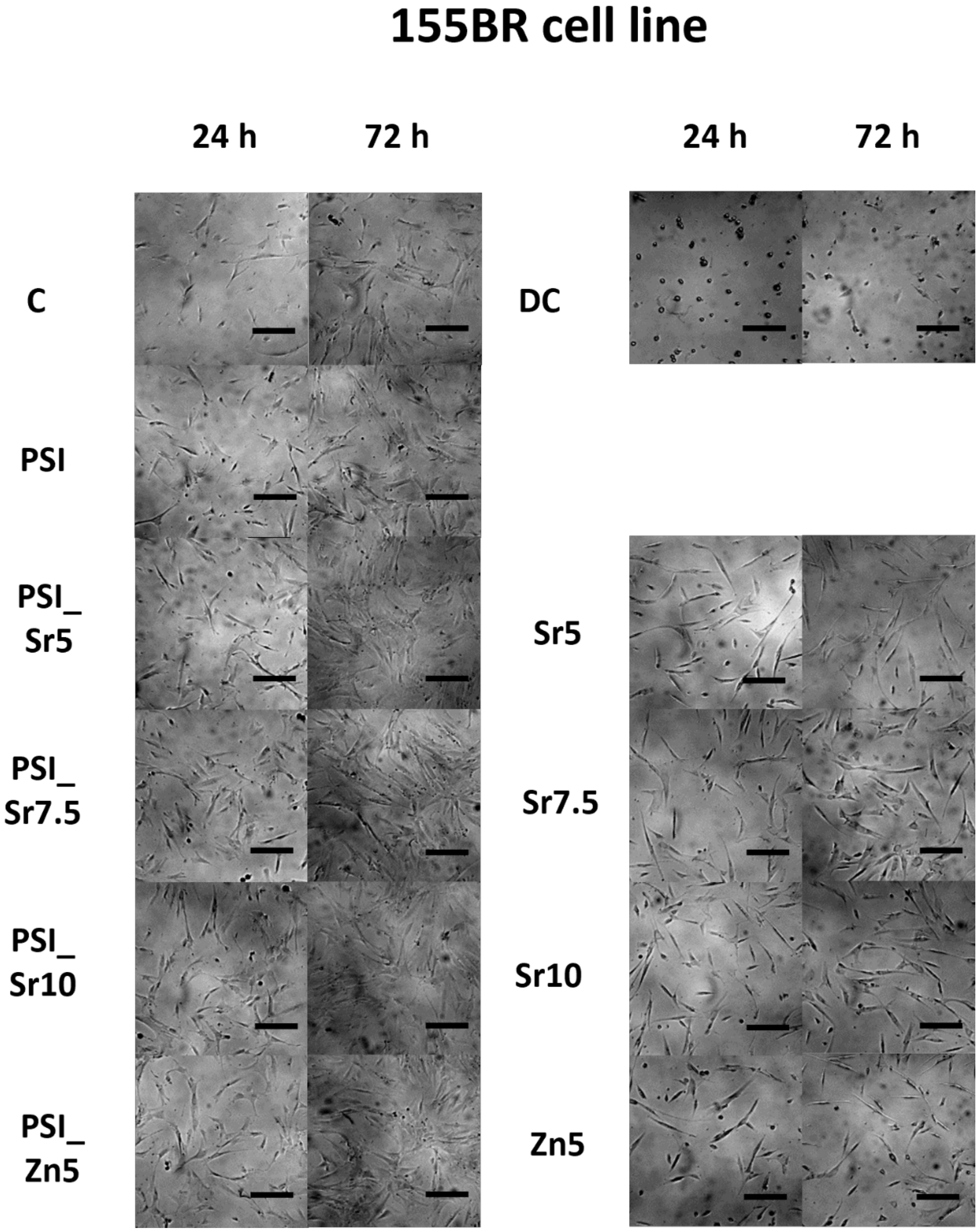

Figure S8: Phase-contrast microscopy images of the 155BR cell line after treatment for 24 and 72 h with different concentrations of the salts and extract of the scaffolds. The scale bars indicate 100 μm.
